# Gastrointestinal stromal tumors in hepatic round ligament cysts: diagnostic utility of detective flow imaging

**DOI:** 10.1055/a-2535-1807

**Published:** 2025-02-26

**Authors:** Akito Furuta, Shunsuke Omoto, Mamoru Takenaka, Michihito Kono, Eisei Nishino, Taro Inoue, Wataru Ono

**Affiliations:** 1 13737 Department of Gastroenterology, Kishiwada Tokushukai Hospital, Osaka, Japan; 2 145700 Department of Gastroenterology, Kobe Tokushukai Hospital, Kobe, Japan; 3 Department of Gastroenterology and Hepatology, Kindai University Faculty of Medicine, Osaka-Sayama, Japan; 4 13737 Department of Pathology, Kishiwada Tokushukai Hospital, Osaka, Japan

Gastrointestinal stromal tumors (GISTs) arising within hepatic round ligament cysts are exceptionally rare, with limited literature available 1 , hence making their definitive diagnosis challenging. Detective flow imaging (DFI) is a novel endoscopic ultrasound (EUS) modality for differentiating malignancies by evaluating irregular vessels 2 3 4 5 . We report a case in which DFI was crucial for differentiating benign and malignant lesions, leading to a diagnosis of GIST within a hepatic round ligament cyst.

A 44-year-old woman was referred for the evaluation of a 30-mm cystic lesion adjacent to the gallbladder. The distinction between the gallbladder and cystic lesions on transabdominal ultrasonography was uncertain. Contrast-enhanced computed tomography revealed a cystic lesion with an enhanced, partially thickened wall ( Fig. 1 ). On magnetic resonance imaging, the gallbladder exhibited a low signal intensity, whereas the cystic lesion demonstrated a high signal intensity on diffusion-weighted image. This indicated that the cyst and gallbladder were discontinuous. EUS revealed a hypoechoic thickened wall in the cystic lesion ( Fig. 2 ). DFI revealed an irregular vessel in the thickened wall within the cystic lesion, suggesting a malignant tumor ( Fig. 3 ). Therefore, we decided to perform surgery ( Video 1 ).

**Fig. 1:**
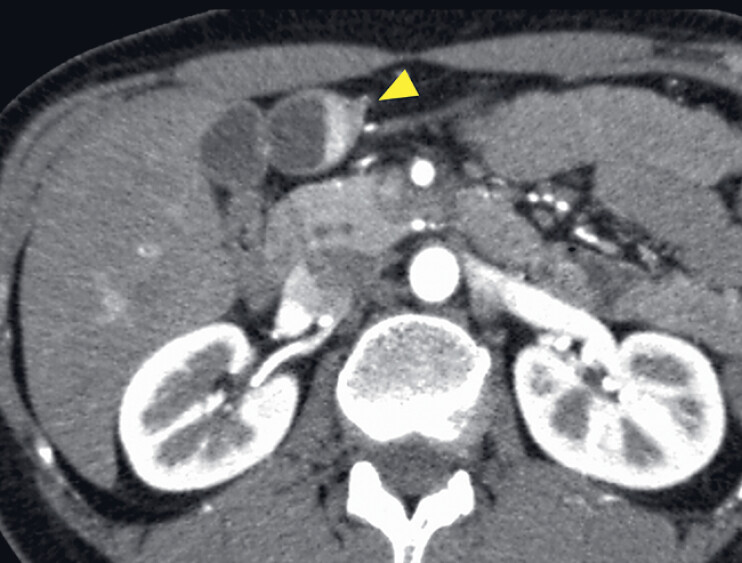
Contrast-enhanced computed tomography reveals a cystic lesion with an enhanced, partially thickened wall (yellow arrowhead) adjacent to the gallbladder.

**Fig. 2:**
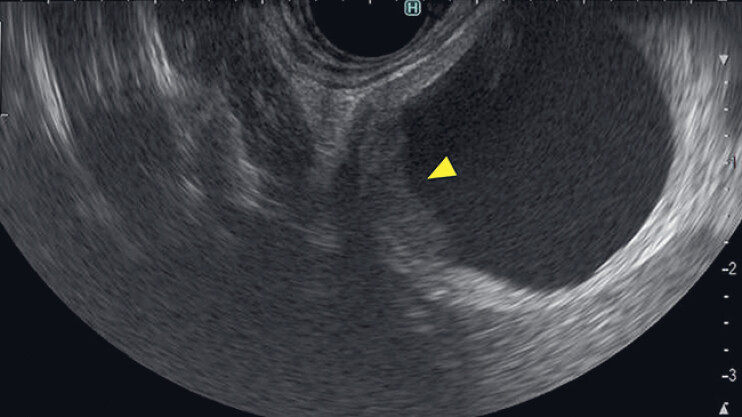
Endoscopic ultrasound shows a hypoechoic thickened wall in the cystic lesion.

**Fig. 3:**
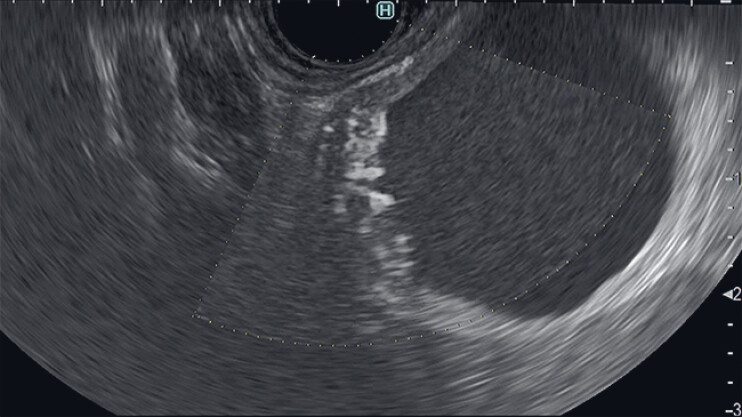
Detective flow imaging shows an irregular vessel in the thickened wall, suggesting a malignant tumor.

A rare gastrointestinal stromal tumor within a hepatic round ligament cyst is diagnosed using detective flow imaging. This novel endoscopic ultrasound technique identifies irregular vessels and guides surgical decision-making.Video 1

During surgery, a cystic tumor was found in the round ligament of the liver. Histopathological examination revealed a cystic tumor with a thickened wall, characterized by sheet-like proliferation of epithelioid cells admixed with spindle-shaped cells, displaying a palisading pattern ( Fig. 4 ). Immunohistochemical staining confirmed the diagnosis of GIST originating from a hepatic round ligament cyst ( Fig. 5 ).

**Fig. 4:**
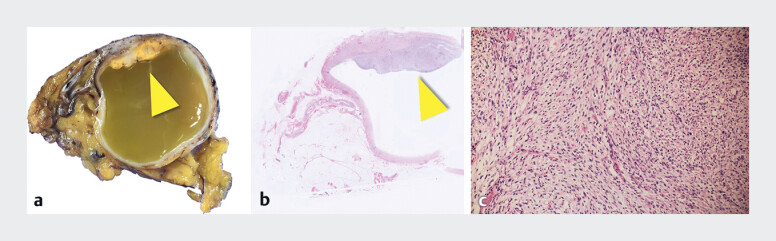
Histopathological examination. **a** Cystic tumor with a thickened wall (yellow arrowhead). **b, c** Hematoxylin-eosin: A thickened wall is characterized by sheet-like proliferation of epithelioid cells admixed with spindle-shaped cells, displaying a palisading pattern.

**Fig. 5:**
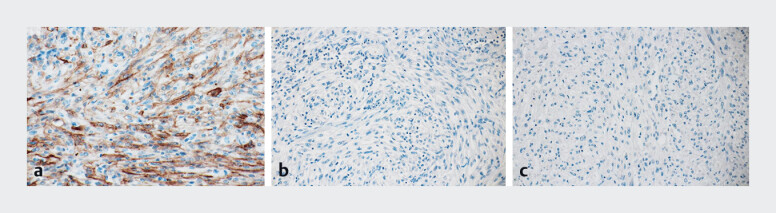
Immunohistochemical staining of the tumor leads to the diagnosis of gastrointestinal stromal tumor. **a** Positive staining for CD117. **b** Negative staining for desmin. **c** Negative staining for S100.

To the best of our knowledge, this is the first reported case using DFI for the evaluation of a GIST arising within a hepatic round ligament cyst. The identification of an irregular vessel on DFI within the tumor can contribute to surgical decision-making in such rare cases.

Endoscopy_UCTN_Code_CCL_1AZ_2AI

## Notes

Endoscopy E-Videos https://eref.thieme.de/e-videos
*E-Videos* is an open access online section of the journal *Endoscopy* , reporting on interesting cases and new techniques in gastroenterological endoscopy. All papers include a high-quality video and are published with a Creative Commons CC-BY license. Endoscopy E-Videos qualify for HINARI discounts and waivers and eligibility is automatically checked during the submission process. We grant 100% waivers to articles whose corresponding authors are based in Group A countries and 50% waivers to those who are based in Group B countries as classified by Research4Life (see: https://www.research4life.org/access/eligibility/ ). This section has its own submission website at https://mc.manuscriptcentral.com/e-videos .
